# Retro-1-Oligonucleotide Conjugates. Synthesis and Biological Evaluation

**DOI:** 10.3390/molecules24030579

**Published:** 2019-02-06

**Authors:** Jordi Agramunt, Enrique Pedroso, Silvia M. Kreda, Rudolph L. Juliano, Anna Grandas

**Affiliations:** 1 Departament de Química Inorgànica i Orgànica (Secció de Química Orgànica) and IBUB, Facultat de Química, Universitat de Barcelona, Martí i Franquès 1-11, 08028 Barcelona, Spain; jagramuntpi@ub.edu (J.A.); epedroso@ub.edu (E.P.); 2 UNC Cystic Fibrosis Center, School of Medicine, University of North Carolina, Chapel Hill, NC 27516, USA; silvia_kreda@med.unc.edu; 3 UNC Eshelman School of Pharmacy, University of North Carolina, Chapel Hill, NC 27599, USA

**Keywords:** Retro-1, oligonucleotide conjugates, antisense, splice switching

## Abstract

Addition of small molecule Retro-1 has been described to enhance antisense and splice switching oligonucleotides. With the aim of assessing the effect of covalently linking Retro-1 to the biologically active oligonucleotide, three different derivatives of Retro-1 were prepared that incorporated a phosphoramidite group, a thiol or a 1,3-diene, respectively. Retro-1–oligonucleotide conjugates were assembled both on-resin (coupling of the phosphoramidite) and from reactions in solution (Michael-type thiol-maleimide reaction and Diels-Alder cycloaddition). Splice switching assays with the resulting conjugates showed that they were active but that they provided little advantage over the unconjugated oligonucleotide in the well-known HeLa Luc705 reporter system.

## 1. Introduction

Forty years after the therapeutic potential of synthetic oligonucleotides was first perceived [1], delivery is the one problem that remains not well solved [2,3]. Oligonucleotides can easily be modified to favor hybridization and ameliorate their bioavailability [4,5], but there is no gold standard methodology for effective and complete internalization. Most forms of oligonucleotides are thought to be taken up by endocytosis and accumulate in intracellular endomembrane compartments [6,7]. Only a tiny fraction of the accumulated oligonucleotide spontaneously leaks from the endosomes, although this is sometimes enough to provide a pharmacological effect. There have been many efforts to increase both the cellular uptake and intracellular release of oligonucleotides including various targeting ligands and endomembrane destabilizing polymers or nanoparticles [2,8].

A few years ago, Juliano and cols. described that the compound named Retro-1 enhanced the pharmaceutical potency of antisense and siRNA oligonucleotides [9]. Retro-1 is a member of a group of compounds able to reduce the action of bacterial toxins [10] by interfering with their intracellular trafficking. Subsequently, high throughput screening has allowed identification of additional molecules capable of enhancing the effectiveness of synthetic oligonucleotides [11,12,13]. Some of the hits developed in these studies have been modified to assess their effect on oligonucleotide delivery [14,15]. However, to our knowledge the impact of covalently linking one of these molecules to the oligonucleotide chain has not been evaluated.

Retro-1, the first compound identified to ameliorate both antisense and siRNA oligonucleotides effect, was chosen for this evaluation. In this manuscript we wish to describe the preparation of three Retro-1 derivatives, their conjugation reactions to a splice switching oligonucleotide, and the results of the biological evaluation. These studies indicate that the efficacy of covalently linking the two moieties is inferior to that achieved by administering the two separate molecules.

## 2. Results and Discussion

### 2.1. Modification of Retro-1 for Conjugation

One alternative to prepare oligonucleotide conjugates, that is, to covalently link oligonucleotides to other molecules, involves the use of a phosphoramidite derivative that can be incorporated into the chain using standard oligonucleotide elongation methodologies. Another possibility is making use of a click reaction [16] that chemoselectively links the two components of the conjugate. Irrespective of the synthesis approach, Retro-1 had to be derivatized so as to incorporate an additional functional group.

**Scheme 1:**
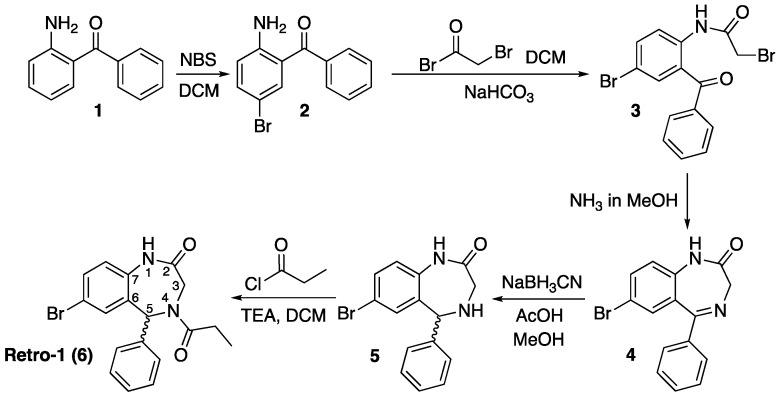
Synthesis scheme described [17] for the preparation of Retro-1, 6. DCM = dichloromethane; NBS = *N*-bromosuccinimide; TEA = triethylamine. The wavy bond indicates that the stereochemistry is not defined (in other words, the compound is a mixture of isomers).

The structure of Retro-1 (**6**), which is shown on Scheme 1, has many points in common with that of benzodiazepine psychoactive drugs. It is also composed of two fused rings, where two atoms of the benzene ring and carbons 6–7 of the diazepine function as bridgehead, and the 1,4-diazepine ring contains a carbonyl group at the 2 position and an aromatic ring appending from carbon 5. The main difference is that atoms 4 and 5 of the diazepine are not linked by a double bond but by a single one. As a result, carbon 5 is a stereocenter.

The synthesis of racemic Retro-1 has been described [17], as well as the separation of the two enantiomers using a chiral HPLC column (conformers, which are observed at the NMR spectra, could not be separated). Both enantiomers were shown to be biologically active, and the difference between their EC_50_ values was small (the *S* isomer was a bit more active than the *R* one, but both were within the same 1–10 μM range as the racemic mixture). Therefore, we have worked with the racemic mixture.

**Scheme 2:**
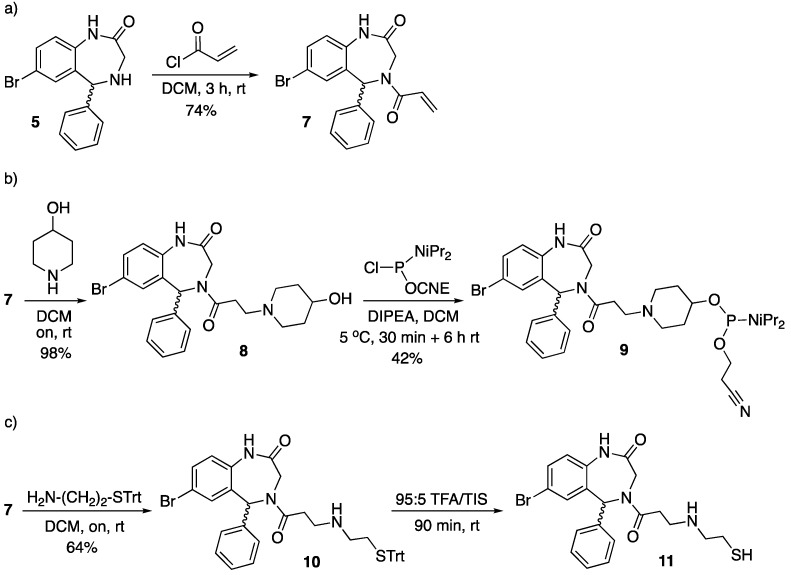
Synthesis of the Retro-1 analogs modified at position 4 of the benzodiazepine ring. The first step (**a**) afforded the common precursor, which was subsequently modified to obtain either phosphoramidite derivative **9** (**b**) or the thiol-modified compound **11** (**c**). CNE = 2-cyanoethyl; DCM = dichloromethane; DIPEA = *N*,*N*-diisopropylethylamine; rt = room temperature; TEA = triethylamine; TFA = trifluoroacetic acid; TIS = triisopropylsilane; Trt = trityl. The wavy bond indicates a not defined stereochemistry.

**Scheme 3:**
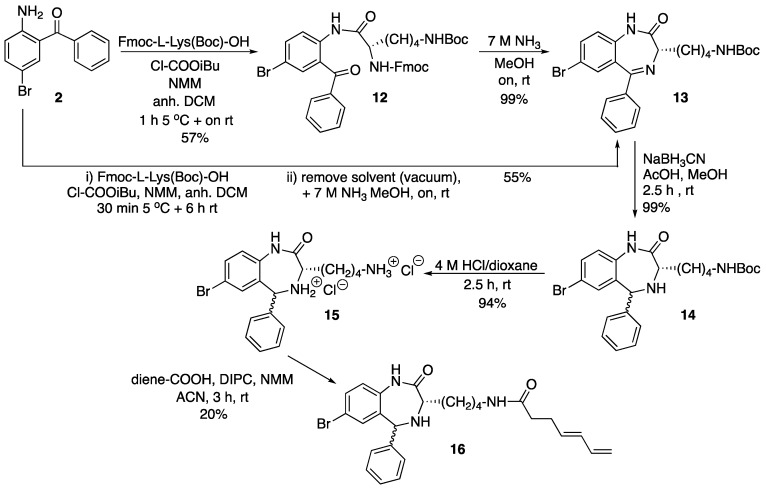
Synthesis of diene-derivatized Retro-1 analog 16. ACN = acetonitrile; Boc = *tert*-butoxycarbonyl; DCM = dichloromethane; diene-COOH = (4*E*)-4,6-heptadienoic acid; DIPC = *N,N*’-diisopropylcarbodiimide; Fmoc = 9-fluorenylmethoxycarbonyl; NMM = *N*-methylmorpholine; on = overnight; rt = room temperature. The wavy bond indicates a not defined stereochemistry.

The only information available on the biological activity of Retro-1 analogs [14] indicates that compounds with no substituent at the 4 position (see Scheme 1) favor oligonucleotide activity, whilst *N*-alkylation seems best suited for reducing bacterial toxins effect. Since Retro-1, in which *N*-4 is acylated, exhibits both effects, we decided to prepare two *N*-4 acylated analogs (Scheme 2) and an additional one modified at the 3 position (Scheme 3).

As shown in Scheme 1, the diazepine ring is made from three building blocks: the aminobenzophenone, the acylating reagent and ammonia. Acylation of the aromatic amine links the two main components and imine formation closes the cycle.

Based on this scheme, which we could perfectly reproduce, changing the reagents of the two acylation reactions appeared to be a simple option to adapt Retro-1 for conjugation. Replacement of bromoacetyl bromide with an activated amino acid would modify position 3 of the diazepine ring, and use of a suitably protected trifunctional amino acid would incorporate an additional group suitable for further derivatization. Alternatively, substitution of propionyl chloride with another acylation reagent would allow position 4 to be modified.

We decided that the three Retro-1 analogs would be differently derivatized for conjugation. One would include a phosphitylatable hydroxyl group, and could be attached to the 5’ end of a resin-linked chain. The other two would contain functional groups suitable for click conjugations in solution. For this purpose we chose a thiol and a 1,3-diene, which were intended to react with a maleimide moiety. Maleimido-oligonucleotides can be easily assembled on a solid support making use of the appropriate maleimide protection [18].

#### 2.1.1. Retro-1 Derivatives Modified at the 4 Position

Two of the three analogs were synthesized from the amine precursor of Retro-1 **5** (see Scheme 1). Replacement of propionyl chloride with acryloyl chloride gave **7** (Scheme 2a), which could be easily modified by means of Michael-type reactions.

Reaction between **7** and 4-hydroxypiperidine afforded **8**, from which phosphoramidite **9** was prepared by reaction with a chlorophosphine and a base (Scheme 2b). The other aza-Michael reaction was carried out with *S*-trityl cysteamine, which furnished **10**, and removal of the trityl group under acidic conditions gave **11** (Scheme 2c). To minimize the extent of thiol oxidation to disulfide the thiol-containing Retro-1 analog was kept protected, and thiol deprotection was carried out not long before the conjugation reaction.

#### 2.1.2. Modification of the 3 Position of Retro-1

The starting material for the preparation of the third Retro-1 derivative was the first intermediate in the synthesis of Retro-1, **2** (see Scheme 1). Acylation of the amine with a suitably protected lysine derivative was found not to be straightforward. Attempts to activate the carboxyl group with either a combination of a carbodiimide and 1-hydroxybenzotriazole, 1-[(1-cyano-2-ethoxy-2-oxoethylideneaminooxy)-dimethylaminomorpholinomethylene)] methan- aminium hexafluorophosphate (COMU), or mesitylenesulfonyl 3-nitro-1,2,4-triazole (MSNT) in the presence of *N*-methylimidazole were unsuccessful. Gratifyingly, the mixed carboxylic carbonic anhydride methodology did work (Scheme 3). Carboxyl group activation with isobutyl chloroformate in the presence of *N*-methylmorpholine afforded **12**, and overnight treatment of **12** with 7 M ammonia in methanol quantitatively removed the Fmoc group and promoted cyclization to **13**. These two steps were also carried out without isolating **12**, and **13** was obtained in a similar yield.

Diene-derivatized **16** was finally obtained after the following series of steps: (i) Reduction of the imine of compound **13** with NaBH_3_CN as previously reported, which gave **14**; (ii) Removal of the Boc group with an acidic treatment, which yielded **15**; (iii) Carbodiimide-promoted acylation of the primary amine with (4*E*)-4,6-heptadienoic acid. We were initially concerned with the possible acylation of the secondary amine (*N*-4 of the diazepine ring), but we found that reaction took place exclusively on the lysine ε-amine, furnishing **16**. In fact, all attempts carried out to acylate *N*-4 failed. Therefore, derivative **16** differed from the other Retro-1 analogs in the presence of a substituent appending from carbon 3 and no acyl group on nitrogen 4.

### 2.2. Preparation of the Retro-1 Conjugates

The three Retro-1 derivatives (**9**, **11**, **16**) were linked to oligonucleotide r5’GTTATTCTTTAGAATGGTGC3’ (all 2’-*O*-methyl and phosphorothioate, T = ribothymidine). This sequence is complementary to that of a mutated intron from thalassemic hemoglobin that is introduced into the firefly luciferase gene used for the splice switching experiments [19]. Compounds **9**, **11** and **16** were also linked to a control oligonucleotide with a scrambled sequence, namely r5’TGTGTACTGATGTAGTTATC3’ (all 2’-*O*-methyl and phosphorothioate; T = ribothymidine).

**Scheme 4:**
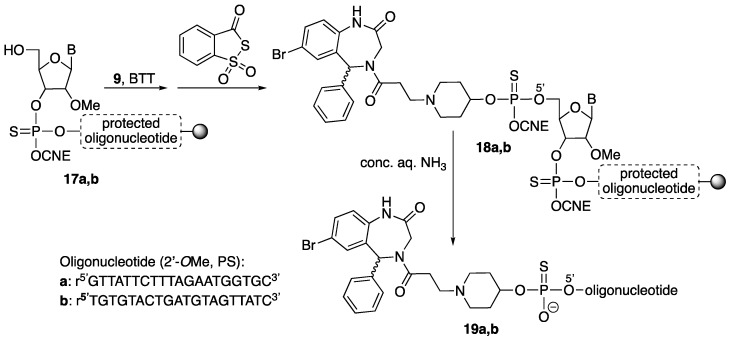
Solid-phase assembly of conjugates **19**. B = G (oligonucleotide a)/T (oligonucleotide b); BTT = 5-benzylthio-1*H*-tetrazole; CNE = 2-cyanoethyl; PS = phosphorothioate. The wavy bond indicates a not defined stereochemistry.

After elongation of the two oligonucleotide chains using standard methodology and BTT as activator (BTT = 5-benzylthio-1*H*-tetrazole), removal of the DMT group (DMT = 4,4’-dimethoxytrityl) on the 5’ end furnished oligonucleotide-resins **17**. This was followed by coupling of phosphoramidite **9** (2 × 10 min coupling, activation with BTT) and sulfurization (Beaucage reagent). The resulting Retro-1-oligonucleotide-resins (**18**) were treated with conc. aq. ammonia (3 h, rt) to yield conjugates **19a** and **19b** (Scheme 4).

**Scheme 5:**
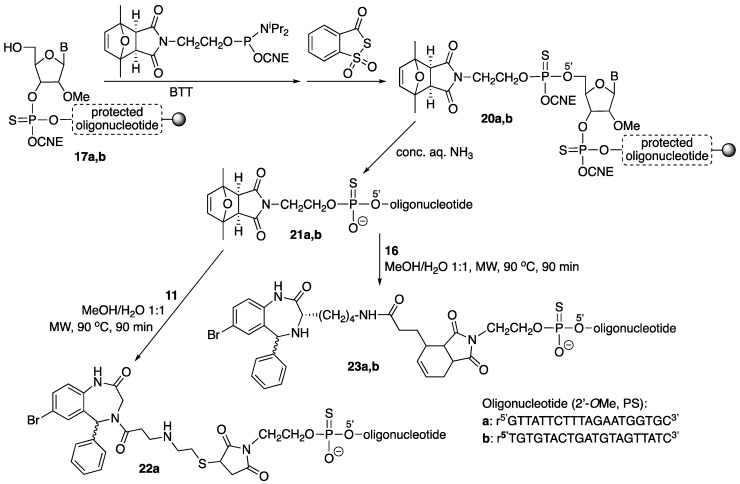
Preparation of conjugates **22** and **23**. B = G (oligonucleotide a)/T (oligonucleotide b); BTT = 5-benzylthio-1*H*-tetrazole; CNE = 2-cyanoethyl; MW = microwave; PS = phosphorothioate. The wavy bond indicates a not defined stereochemistry.

For the preparation of the other conjugates (Scheme 5), the 2,5-dimethylfuran-protected maleimide phosphoramidite was coupled to either oligonucleotide-resin **17** (2 × 10 min coupling, activation with BTT), and the resulting phosphite triester sulfurized (Beaucage reagent). Treatment of oligonucleotide-reins **20** with conc. aq. ammonia afforded [protected maleimido]-oligonucleotides **21**, which were purified. Conjugates **22** and **23** were obtained by simultaneously carrying out maleimide deprotection (retro Diels-Alder reaction) and reaction with either the thiol-containing derivative **11** (Michael-type reaction) or the diene-derivatized compound **16** (Diels-Alder cycloaddition) in a microwave (MW) oven.

All conjugates were purified by reversed phase HPLC and characterized by MALDI-TOF MS.

### 2.3. Splice-Switching Assays

**Figure 1:**
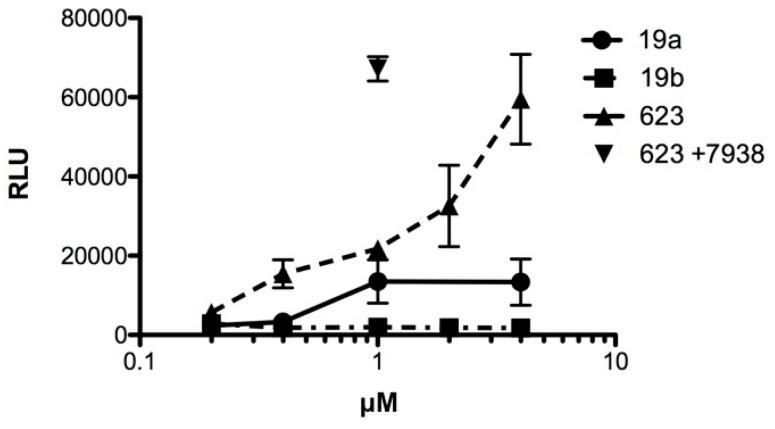
HeLa705 cells in complete growth medium were incubated for 16 h with various concentrations of conjugated or unconjugated oligonucleotide. Thereafter the cells were recovered and assayed for luciferase activity. In one case cells were post-treated with 10 μM UNC7938 for 4 h after exposure to SSO623 and then assayed. RLU = relative luminescence units; n = 3. Means and standard errors shown. SSO623: r5’GTTATTCTTTAGAATGGTGC3’, all 2’-*O*-Me and phosphorothioate (PS) (T = ribothymidine).

Oligonucleotides conjugated to Retro-1 derivatives were examined for splice correction activity. The experiments utilized the HeLa Luc 705 cell line [19], as described in Materials and Methods. Oligonucleotides having the known splice correcting sequence r5’GTTATTCTTTAGAATGGTGC3’, previously termed SSO623 [19], were used in conjugated or unconjugated form and were compared to control oligonucleotides with the inactive sequence r5’TGTGTACTGATGTAGTTATC3’. In Figure 1 oligonucleotide **19a**, which is the immediate conjugate of Retro-1, was compared to its control conjugate **19b**. Furthermore, we examined unmodified SSO623, as well as SSO623 followed by treatment with the small molecule UNC7938 as a positive control. As seen in the figure, oligonucleotide **19a** produced a dose-dependent luciferase induction while the control oligonucleotide **19b** did not. However, SSO623 itself also gave rise to a progressive induction of luciferase that was somewhat greater than that produced by **19a**. The dual use of SSO623 and UNC7938 provided the largest degree of splice correction and luciferase induction as expected. This set of experiments demonstrated that coupling of the Retro moiety did not prevent the splice correcting activity of the active oligonucleotide. However, conjugate **19a** did not provide an advantage, in terms of potency or efficacy, over SSO623 itself. We also examined two additional conjugates **22a** and **23a**. However, neither of these conjugates displayed an advantage over SSO623 in the HeLa Luc 705 induction assay.

In summary, conjugation of the Retro moiety to a splice switching oligonucleotide did not provide a major enhancement of splice correction activity in the widely used HeLa Luc705 reporter system. At this point it is unclear why the conjugates displayed slightly lower activity than the unmodified oligonucleotide. One could hypothesize that the presence of the bulky Retro group might affect either cell uptake of the oligo or might affect the interaction of the oligo with the splicing machinery. However, detailed investigation of these possibilities, especially the splicing aspect, would involve a very substantial amount of new biological investigation and is beyond the scope of this work.

## 3. Experimental Section

### 3.1. Materials and Methods

#### 3.1.1. Materials for Solution Organic Synthesis

(*E*)-Hepta-4,6-dienoic acid was prepared as described by Baillie et al. [20], and *S*-trityl cysteamine as reported by Naumiec et al. [21]. Fmoc-l-Lys(Boc)-OH was from Novabiochem (Zaragoza, Spain); all of the other chemicals were either from Sigma-Aldrich (Zaragoza, Spain) or Across Organics (Zaragoza, Spain) (7 M ammonia in methanol), and were used without further purification. Water was obtained from a MilliQ system (Zaragoza, Spain).

#### 3.1.2. Analysis and Characterization Techniques for Small Organic Molecules (**2**–**16**)

TLC was carried out on silica gel plates 60 F254 from Merck (Zaragoza, Spain). IR spectra were recorded in a Nicolet 6700 FT-IR spectrometer (Thermo Scientific, Zaragoza, Spain). ^1^H and ^13^C NMR spectra were recorded on either Varian Mercury 400 MHz or Brucker 400 MHz spectrometers (reference: TMS or residual solvent signals). HR-ESI mass spectra were obtained using an LC/MSD-TOF Instrument (Agilent Technologies, Zaragoza, Spain). HPLC/MS analyses were recorded in an Alliance Waters 2690 separation module with a Waters micromass ZQ4000 MS detector (Waters, Zaragoza, Spain). Aqueous solutions were lyophilized in either Labconco (Vertex, Zaragoza, Spain) or Christ freeze dryers (Inycom, Zaragoza, Spain).

#### 3.1.3. Materials for Oligonucleotide Assembly

Nucleoside phosphoramidites (2′-*O*Me A^Pac^, C^Ac^, G^iPrPac^, and 5-MeU), 5′-*O*-DMT-2′-*O*Me-C^Ac^-succinyl-derivatized glass beads, and oligonucleotide synthesis reagents were from Link Technologies (Manchester, UK). [Protected maleimido]-phosphoramidite (see structure in Scheme 5) was synthesized as previously described [18].

#### 3.1.4. Oligonucleotide Assembly

Oligonucleotide chains were elongated in a 3400 ABI automatic synthesizer at the 1 μmol scale, using standard phosphite triester methodology. The activator used at the coupling step was 5-benzylthio-1*H*-tetrazole (0.3 M solution in anh. acetonitrile, 10 min coupling time), and the Beaucage reagent was used for sulfurization (0.05 M in anh. acetonitrile; 2 × 4 min). All phosphoramidites were dissolved in anh. acetonitrile (0.1 M solutions), except that of 2′-*O*Me-5-MeU (which was dissolved in anh. DCM).

Final deprotection conditions: conc. aq. ammonia, 3 h, room temperature. After this treatment the resin was filtered and washed with water (3×), the filtrates pooled and concentrated in a SpeedVac apparatus (Inycom, Zaragoza, Spain) (removal of ammonia), and the resulting crude lyophilized.

#### 3.1.5. RP-HPLC Analysis and Purification of Oligonucleotides and Conjugates

Reversed-phase HPLC analysis and purification was performed using a Shimadzu system (Izasa, Zaragoza, Spain). Linear gradients were always used.

Analysis conditions for oligonucleotides and conjugates were Jupiter C18 column (10 μm, 300 Å, 250 × 4.6 mm) from Phenomenex (Zaragoza, Spain), solvent A: 0.1 M aq. triethylammonium acetate, solvent B: acetonitrile, flow: 1 mL/min, detection wavelength: 254 nm. Semipreparative purification conditions: Jupiter C18 column (10 μm, 300 Å, 250 × 10.0 mm) from Phenomenex, solvent A: 0.1 M aq. triethylammonium acetate, solvent B: acetonitrile, flow: 3 mL/min, detection wavelength: 254 nm.

#### 3.1.6. MALDI-TOF MS of Oligonucleotides and Conjugates

MALDI-TOF mass spectra were recorded on a 4800 Plus instrument (AB Sciex, Millipore, Spain), not using the reflector mode unless otherwise indicated. Typical analysis conditions: 1:1 (*v*/*v*) 2,4,6-trihydroxyacetophenone/ammonium citrate (THAP/CA), negative mode.

### 3.2. Synthesis of Small Molecules (Compounds ***8**–**16***)

Compounds **1**–**6** were Synthesized as Previously Described [17].

*7-Bromo-1,3,4,5-tetrahydro-4-(acryloyl)-5-phenyl-2H-1,4-benzodiazepin-2-one* (**7**). To a suspension of **5** (150 mg, 0.47 mmol) in DCM (5 mL) acryloyl chloride (50 µL, 0.62 mmol) was added, and the mixture stirred at room temperature for 3 h. Afterwards, additional DCM (30 mL) was added, and the mixture was transferred to a separatory funnel and washed with H_2_O (3 × 20 mL). The organic layer was dried over anh. MgSO_4_, filtered, and the solvent removed under low pressure. The resulting crude was purified by silica gel flash column chromatography eluting with DCM/EtOAc mixtures from 100:0 to 75:25. The title compound (**7**) was obtained as a white foam (130 mg, 74%).

TLC (DCM/EtOAc 80:20): R*_f_* = 0.50; IR (ATR, solid): 3221, 2920, 2359, 2340, 1682, 1647, 1515, 1416, 665 cm^−1^; ^1^H NMR (CDCl_3_, 400 MHz): δ 8.88 and 8.85 (s, 1H), 7.45 (s, 1H), 7.46 and 7.40 (d, *J* = 8.5 Hz, 1H), 7.32–7.24 (m, 3H), 7.05 (s, 1H), 7.04 and 6.16 (s, 1H), 6.93 and 6.89 (d, *J* = 8.5 Hz, 1H), 6.61 and 6.54 (dd, *J =* 10.4, 16.4 Hz, 1H), 6.45 and 6.41 (s, 1H), 5.84–5.78 (m, 1H), 4.34–4.02 (m, 2H) ppm; diastereomer 1, ^13^C NMR (CDCl_3_, 101 MHz): δ 170.39, 166.04, 138.44, 134.30, 132.09, 130.61, 129.02, 127.93, 126.67, 122.91, 117.43, 59.46, 48.72 ppm; diastereomer 2, ^13^C NMR (CDCl_3_, 101 MHz): δ 170.39, 166.63, 137.52, 135.52, 134.71, 133.15, 132.73, 130.86, 130.00, 129.06, 128.39, 128.21, 127.08, 126.79, 123.67, 117.55, 63.49, 46.30 ppm; ESI-HRMS (positive mode): *m/z* 371.0391/373.0368 (^81^Br) [M + H]^+^, M calcd for C_18_H_16_BrN_2_O_2_ 371.0390.

*7-Bromo-1,3,4,5-tetrahydro-4-[1-oxo-3-(4-hydroxypiperidin-1-yl)propyl]-5-phenyl-2H-1,4-benzodiazep-in-2-one* (**8**). **7** (100 mg, 0.27 mmol) and 4-hydroxypiperidine (273.1 mg, 2.70 mmol) were dissolved in DCM (5 mL) and reacted overnight at room temperature. Afterwards, the solvent was removed under low pressure, the resulting crude dissolved in EtOAc (40 mL) and washed with H_2_O (3 × 20 mL). The organic phase was dried over anh. MgSO_4_, filtered, and the solvent removed under vacuum. The title compound (**8**) was obtained as a white solid (125 mg, 98%).

TLC (DCM:EtOAc 50:50): R*_f_* = 0.20; IR (ATR, solid): 3214, 3119, 2983, 2353, 2334, 1865, 1650, 1558, 1553, 1508, 1239, 783, 666 cm^−1^; ^1^H NMR (CDCl_3_, 400 MHz): δ 8.70 and 8.68 (s, 1H), 7.46–7.38 (m, 2H), 7.34–7.27 (m, 3H), 7.06–7.00 (m, 2H), 6.95 and 6.20 (s, 1H), 6.92–6.87 (m, 1H), 4.45–3.98 (m, 2H), 3.65 (p, *J* = 4.5 Hz, 1H), 2.80–2.54 (m, 6H), 2.20–2.12 (m, 2H), 1.87–1.79 (m, 4H), 1.57–1.47 (m, 1H) ppm; diastereomer 1, ^13^C NMR (CDCl_3_, 101 MHz): δ 171.64, 170.40, 138.33, 134.86, 134.25, 132.05, 130.34, 129.14, 128.96, 127.74, 122.87, 117.39, 67.49, 63.07, 59.29, 53.75, 51.19, 34.18, 31.60 ppm; diastereomer 2, ^13^C NMR (CDCl_3_, 101 MHz): δ 171.96, 170.94, 137.60, 135.50, 133.17, 132.60, 130.37, 128.44, 128.10, 127.05, 123.45, 117.19, 67.44, 60.42, 53.82, 51.30, 46.10, 34.19, 31.30 ppm; ESI-HRMS (positive mode): *m/z* 472.1222/474.1205 (^81^Br) [M + H]^+^, M calcd for C_23_H_27_BrN_3_O_3_ 472.1230.

*7-Bromo-1,3,4,5-tetrahydro-4-[1-oxo-3-(4-hydroxypiperidin-1-yl)propyl]-5-phenyl-2H-1,4-benz-odiazepin-2-one, 2-cyanoethyl N,N-diisopropylphosphoramidite* (**9**). **8** (453.5 mg, 0.96 mmol) was dissolved in anh. DCM (15 mL). Subsequently, anh. *N*,*N*-diisopropylethylamine (310 µL, 2.41 mmol) was added and the mixture was stirred in an ice bath for 5 min under an argon atmosphere. Afterwards, a solution of chloro(2-cyanoethoxy)diisopropylaminophosphine (250 mg, 1.06 mmol) in anh. DCM (1 mL) was added, and the mixture was reacted in an ice bath for 30 min. Then, it was allowed to warm up and left stirring for 6 h at room temperature until complete phosphitylation as shown by TLC. The solvent was removed under low pressure; the crude was dissolved in EtOAc (50 mL) and washed with aq. NaHCO_3(sat.)_ (3 × 30 mL). The organic phase was dried over anh. MgSO_4_, filtered, and the solvent removed under low pressure. The resulting crude was further purified by silica gel flash column chromatography eluting with DCM/EtOAc/NEt_3_ mixtures from 98:0:2 to 48:50:2. The title compound (**9**) was obtained as a white foam (272.4 mg, 42%).

TLC (DCM/EtOAc/NEt_3_ 48:50:2): R*_f_* = 0.50; ^1^H NMR (CDCl_3_, 400 MHz): δ 7.90 and 7.86 (s, 1H), 7.48–7.40 (m, 2H), 7.33–7.27 (m, 3H), 7.05–7.01 (m, 2H), 6.95 and 6.22 (s, 1H), 6.88–6.80 (m, 1H), 4.44–4.00 (m, 2H), 3.90–3.71 (m, 3H), 3.65–3.45 (m, 2H), 2.80–2.56 (m, 8H), 2.38–2.23 (m, 2H), 1.93–1.79 (m, 2H), 1.76–1.60 (m, 2H), 1.27 and 1.17 (dd, *J* = 6.8, 5.5 Hz, 12H) ppm; ^31^P NMR (CDCl_3_, 162 MHz): δ 145.85 ppm; ESI-HRMS (positive mode): *m/z* 672.2298/674.2281 (^81^Br) [M + H]^+^, M calcd for C_32_H_44_BrN_5_O_4_P 672.2309.

*7-Bromo-5-phenyl-4-{3-[(2-(tritylthio)ethyl)amino]propanoyl}-1,3,4,5-tetrahydro-2H-benzo[e][1,4]di-azepin-2-one* (**10**). **7** (370 mg, 0.99 mmol) and *S*-tritylcysteamine (1.50 g, 4.49 mmol) were dissolved in DCM (5 mL) and reacted overnight at room temperature. Afterwards, the solvent was removed under low pressure, and the resulting crude was purified by silica gel flash column chromatography eluting with hexanes/EtOAc/MeOH mixtures from 30:70:0 to 0:100:3. The title compound (**10**) was obtained as a white solid (441 mg, 64%).

TLC (EtOAc/MeOH 97:3): R*_f_* = 0.35; IR (ATR, solid): 3211, 2926, 1729, 1666, 1664, 1482, 1448, 1368, 1242, 1216, 1188, 1058, 821, 745, 694 cm^−1^; ^1^H NMR (CDCl_3_, 400 MHz): δ 7.44–7.40 (m, 9H), 7.30–7.18 (m, 14H), 7.03–6.99 (m, 1H), 6.90 and 6.11 (s, 1H), 4.42–3.96 (m, 2H), 2.80–2.66 (m, 2H), 2.60–2.45 (m, 3H), 2.41–2.31 (m, 3H), 1.87 (br s, 1H) ppm; diastereomer 1, ^13^C NMR (CDCl_3_, 101 MHz): δ 171.49, 169.75, 145.01, 138.34, 134.80, 134.45, 133.43, 132.28, 130.38, 129.15, 128.00, 127.85, 127.13, 126.80, 122.89, 117.61, 66.71, 63.16, 48.73, 44.67, 41.11, 33.67, 31.86 ppm; diastereomer 2, ^13^C NMR (CDCl_3_, 101 MHz): δ 171.86, 170.35, 144.90, 137.43, 135.47, 133.43, 132.83, 130.41, 129.33, 128.62, 128.30, 128.04, 127.13, 126.80, 123.40, 117.61, 66.80, 63.16, 48.40, 44.89, 41.11, 33.31, 31.81 ppm; ESI-HRMS (positive mode): *m/z* 690.1776/692.1765 (^81^Br) [M + H]^+^, M calcd for C_39_H_37_BrN_3_O_2_S 690.1784.

**Figure 2:**
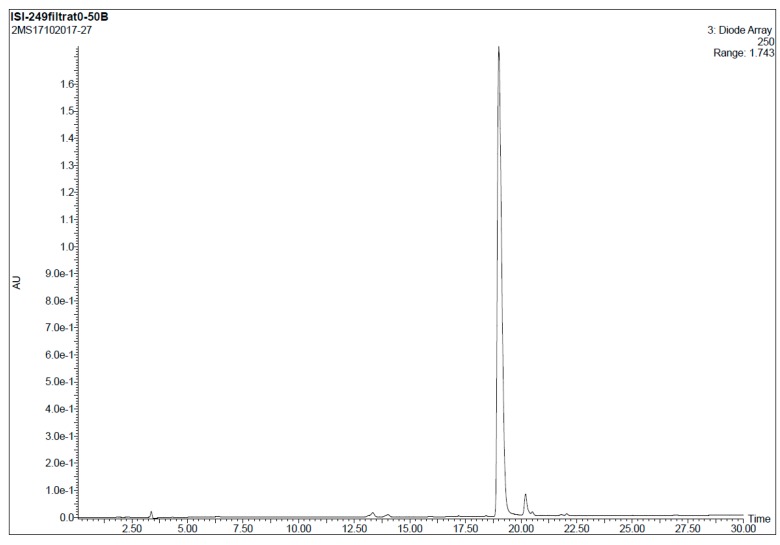
Analytical HPLC trace of crude 11 (detection wavelength: 250 nm).

*7-Bromo-4-{3-[(2-mercaptoethyl)amino]propanoyl}-5-phenyl-1,3,4,5-tetrahydro-2H-benzo[e][1,4]diaze-pin-2-one* (**11**). **10** (20 mg, 0.03 mmol) was dissolved in a mixture of TFA/TIS (95:5) and reacted at room temperature for 90 min. Afterwards, the solvent was removed under a N_2_ stream and the resulting crude dissolved in a mixture MeOH/H_2_O (1:1, 2 mL) and filtered through a hydrophilic PTFE (polytetrafluoroethylene) syringe filter (0.22 µm), lyophilized and used without further purification (quantitative thiol deprotection as assessed by HPLC, Figure 2).

HPLC-MS: Analysis conditions: 0 → 50 % B in 30 min, t_R_ = 19.0 min. ESI-MS (positive mode) of the main peak: *m/z* 448.2/450.2 (^81^Br), M calcd for C_20_H_23_BrN_3_O_2_S 448.07. 

*N-(N^α^-Fmoc-N^ε^-l-lysinyl)-2-amino-5-bromobenzophenone* (**12**). *N*-Methylmorpholine (200 µL, 1.81 mmol) and isobutylchloroformate (94 µL, 0.73 mmol) were added to a solution of Fmoc-l-Lys(Boc)-OH (340.5 mg, 0.73 mmol) in anh. DCM (5 mL), and the mixture cooled in an ice bath. After 15 min, a solution of **2** (200.0 mg, 0.73 mmol) in anh. DCM (1 mL) was added and the mixture was stirred for 1 h at 5 °C and overnight at room temperature. Afterwards, further DCM was added (15 mL) and the mixture was washed with aq. HCl 10% (2 × 10 mL). The organic phase was dried over anh. MgSO_4_, filtered and the solvent evaporated under low vacuum. The resulting crude was purified by silica gel flash column chromatography eluting with DCM/MeOH mixtures from 100:0 to 97:3. The title compound (**12**) was obtained as a pale white foam (301 mg, 57%).

TLC (DCM/MeOH 95:5): R*_f_* = 0.40; IR (ATR, solid): 2980, 2905, 2355, 1730, 1658, 1599, 1571, 1497, 1425, 1437, 1391, 1254, 1248, 1173, 1158, 1071, 1043, 757, 744, 694, 533 cm^−1^; ^1^H NMR (CDCl_3_, 400 MHz): δ 8.57 (d, *J* = 8.8 Hz, 1H), 7.85–7.52 (m, 9H), 7.46–7.23 (m, 7H), 5.95 (br. s, 1H), 4.78–4.63 (m, 1H), 4.55–4.34 (m, 2H), 4.32–4.20 (m, 2H), 3.13 (t, *J* = 8.0 Hz, 2H), 2.02 (m, 2H), 1.93–1.78 (m, 2H), 1.77–1.50 (m, 2H), 1.45 (s, 9H) ppm; ^13^C NMR (CDCl_3_, 101 MHz): δ 197.84, 171.21, 156.47, 156.31, 143.69, 141.23, 138.78, 137.60, 136.75, 135.51, 132.91, 129.94, 128.46, 127.73, 127.66, 127.09, 127.05, 125.47, 125.14, 120.01, 119.89, 115.13, 79.21, 77.48, 77.16, 76.84, 67.44, 56.46, 47.20, 39.77, 31.73, 29.81, 28.47, 22.42 ppm. ESI-HRMS (positive mode): *m/z* 726.2173/728.2161 (^81^Br) [M + H]^+^; M calcd for C_39_H_40_BrN_3_O_6_ 726.2173.

*Tert-Butyl (S)-[4-(7-bromo-2-oxo-5-phenyl-2,3-dihydro-1H-benzo[e][1,4]diazepin-3-yl)butyl]carbamate* (**13**). **12** (150 mg, 0.21 mmol) was dissolved in a 7 M ammonia solution (in MeOH, 3 mL) and reacted at room temperature overnight. Afterwards, the reaction mixture was taken to dryness and the crude purified by silica gel flash column chromatography eluting with a 70:30 hexanes/EtOAc mixture. The title compound (**13**) was obtained as a pale yellow solid (104 mg, 99%). For characterization see below.

*One-pot synthesis of compound***13***from***2**. A solution of Fmoc-l-Lys(Boc)-OH (1.87 g, 4.00 mmol) in anh. DCM (15 mL) was cooled in an ice bath. *N*-Methylmorpholine (1.37 mL, 9.09 mmol) and isobutyl chloroformate (0.81 mL, 3.64 mmol) were added. After 10 min, a solution of **2** (1.00 g, 3.64 mmol) in anh. DCM (2 mL) was added, and the mixture was stirred for 30 min at 5 °C and 5.5 h at room temperature. Afterwards, the solvent was removed under vacuum and the crude dissolved in 7 M NH_3_ (in MeOH, 40 mL), and the solution was left to react overnight at room temperature. Subsequently, the solvent was removed under vacuum and the resulting crude dissolved in EtOAc (100 mL) and washed with 10% aq. HCl (3 × 30 mL). The organic phase was dried over anh. MgSO_4_, filtered and the solvent evaporated under vacuum. The crude material was further purified by silica gel flash column chromatography eluting with a 70:30 hexanes/EtOAc mixture. The title compound (**13**) was obtained as a pale yellow solid (970 mg, 55%).

TLC (hexanes/EtOAc 1:1): R*_f_* = 0.42; IR (ATR, solid): 3414, 3309, 2929, 2359, 2337, 1682, 1653, 1508, 1232, 1156, 669 cm^−1^; ^1^H NMR (CDCl_3_, 400 MHz): δ 9.45 (s, 1H), 7.60 (dd, *J* = 8.6, 2.3 Hz, 1H), 7.52–7.34 (m, 6H), 7.08 (d, *J* = 8.6 Hz, 1H), 4.63 (s br, 1H), 3.50 (dd, *J* = 8.2, 5.7 Hz, 1H), 3.19 (d, *J* = 7.6 Hz, 2H), 2.32–2.14 (m, 2H), 1.69–1.56 (m, 2H), 1.44 (s, 11H) ppm; ^13^C NMR (CDCl_3_, 101 MHz): δ 172.13, 168.09, 156.03, 138.64, 137.65, 134.57, 133.30, 130.51, 129.67, 129.10, 128.34, 123.04, 116.00, 79.01, 63.22, 40.47, 30.66, 30.00, 28.44, 23.32 ppm; ESI-HRMS (positive mode): *m/z* 486.1386/488.1370 (^81^Br) [M + H]^+^, M calcd for C_24_H_29_BrN_3_O_3_ 486.1387.

*7-Bromo-1,3,4,5-tetrahydro-4-[4-(N-Boc-amino)butyl]-5-phenyl-2H-1,4-benzodiazepin-2-one* (**14**). To a solution of **13** (800 mg, 1.65 mmol) and NaBH_3_CN (155.4 mg, 2.48 mmol) in MeOH (10 mL), AcOH (500 µL, 8.24 mmol) was added, and the mixture was stirred at room temperature for 2.5 h until complete reduction of the imine as assessed by TLC. Afterwards, the solvent was removed under low pressure, the crude was dissolved in EtOAc (50 mL) and washed with aq. NaHCO_3(sat)_ (2 × 20 mL). The organic layer was dried over anh. MgSO_4_, filtered and the solvent removed under low pressure. The title compound (**14**) was obtained as a pale yellow solid (799 mg, 99%).

TLC (hexanes/EtOAc 1:1): R*_f_* = 0.52; IR (ATR, solid): 3290, 2967, 2929, 2866, 1663, 1479, 1365, 1245, 1159, 817, 700 cm^−1^; ^1^H NMR (CDCl_3_, 400 MHz): δ 7.99 and 7.67 (s, 1H), 7.45–7.27 (m, 5H), 7.13 and 6.76 (d, *J* = 2.3 Hz, 1H), 6.89 and 6.80 (d, 8.4 Hz, 1H), 5.34 and 5.24 (s, 1H), 4.56 (br s, 1H), 3.57 and 3.31 (t, *J* = 7.2 Hz, 1H), 3.14–3.05 (m, 2H), 1.94–1.44 (m, 6H), 1.42 (s, 9H) ppm; diastereomer 1, ^13^C NMR (CDCl_3_, 101 MHz): δ 175.02, 156.00, 142.23, 139.97, 136.56, 132.23, 128.64, 128.45, 127.19, 122.69, 117.53, 79.06, 63.98, 59.94, 40.29, 31.65, 29.93, 28.41, 23.50 ppm; diastereomer 2, ^13^C NMR (CDCl_3_, 101 MHz): δ 174.27, 156.00, 142.33, 139.97, 135.88, 131.35, 128.57, 128.20, 127.54, 123.32, 118.88, 79.06, 58.97, 56.14, 40.29 31.65, 30.04, 28.42, 23.21 ppm; ESI-HRMS (positive mode): *m/z* 488.1559/490.1555 (^81^Br) [M + H]^+^, M calcd for C_24_H_31_BrN_3_O_3_ 488.1543.

*7-Bromo-1,3,4,5-tetrahydro-4-(4-aminobutyl)-5-phenyl-2H-1,4-benzodiazepin-2-one dihydrochloride* (**15**). In a 10 mL round-bottom flask, **14** (100 mg, 0.21 mmol) was dissolved in 4 M HCl (in dioxane, 5 mL) and the reaction mixture was left stirring for 2.5 h at room temperature. Afterwards the solvent was removed under low pressure to afford the title product (**15**) as a pale yellow solid (94 mg, 99%).

TLC (DCM/MeOH 9:1): R*_f_* = 0.20; IR (ATR, solid): 3474, 3199, 2911, 2711, 2524, 1704, 1590, 1568, 1479, 1448, 1375, 1315, 1270, 1245, 1131, 998, 916, 827, 697 cm^−1^; ^1^H NMR (CD_3_OD_,_ 400 MHz): δ 7.75 and 7.73 (s, 1H), 7.69–7.55 (m, 4H), 7.44–7.38 (m, 1H), 7.34–7.29 (m, 2H), 7.19 (d, *J* = 8.5 Hz) and 7.09 (d, *J* = 9.2 Hz, 1H), 6.02 and 5.71 (s, 1H), 4.12 (dd, *J* = 9.0, 4.6 Hz) and 3.86 (dd, *J* = 10.5, 3.1 Hz, 1H), 2.99–2.89 (m, 2H), 2.32–2.10 (m, 1H), 2.00–1.80 (m, 1H), 1.71 (h, *J* = 7.3 Hz, 2H), 1.58–1.34 (m, 2H) ppm; diastereomer 1, ^13^C NMR (CDCl_3_, 101 MHz): δ 166.45, 138.28, 135.43, 135.17, 133.98, 130.67, 130.20, 129.58, 130.20, 129.58, 128.34, 125.45, 120.24, 60.83, 57.09, 40.24, 28.13, 27.96, 23.88 ppm; diastereomer 2, ^13^C NMR (CDCl_3_, 101 MHz): δ 167.82, 136.80, 135.87, 134.92, 133.34, 131.13, 130.35, 129.58, 128.34, 126.16, 119.84, 63.60, 57.38, 40.24, 29.17, 28.05, 23.66 ppm; ESI-HRMS (positive mode): *m/z* 388.1016/390.1000 (^81^Br) [M + H]^+^, M calcd for C_19_H_23_BrN_3_O 388.1019.

*7-Bromo-1,3,4,5-tetrahydro-4-{4-[N-[(4E)-4,6-heptadienoyl]-amino]butyl}-5-phenyl-2H-1,4-benzodiazepin-2-one* (**16**). *N*,*N’*-Diisopropylcarbodiimide (61 µL, 0.39 mmol) and *N*-methylmorpholine (2.6 µL, 0.03 mmol) were added to a solution of (4*E*)-4,6-heptadienoic acid (49 mg, 0.39 mmol) in HPLC-quality acetonitrile (1 mL), and the mixture was left stirring for 10 min. Afterwards, **15** (50 mg, 0.13 mmol) and an additional amount of *N*-methylmorpholine (2.6 µL, 0.03 mmol) were poured onto the mixture. Additional amounts of *N*-methylmorpholine (2.6 µL, 0.03 mmol) were added after 20 and 40 min, respectively, and the solution was stirred for up to 2 h at room temperature. Subsequently, acetonitrile was added (4 mL), and the crude was filtered using a hydrophilic PTFE syringe filter (0.22 µm), purified by RP-HPLC (Jupiter Proteo C18 (10 μm, 250 nm, 250 × 10 mm) from Phenomenex, solvent A: H_2_O 0.1% formic acid; solvent B: ACN 0.1% formic acid, linear gradient 40 → 80% B, 3 mL/min, detection wavelength 254 nm) and lyophilized. The title compound (**16**) was obtained as a white solid (10.7 mg, 20%).

TLC (EtOAc/MeOH 9:1): R*_f_* = 0.10; IR (ATR, solid): 2980, 2905, 2355, 1730, 1658, 1599, 1571, 1497, 1425, 1437, 1391, 1254, 1248, 1173, 1158, 1071, 1043, 757, 744, 694, 533 cm^−1^; ^1^H NMR (CDCl_3_, 400 MHz) δ 7.65–7.53 (m, 4H), 7.42–7.28 (m, 4H), 7.16 and 7.04 (d, *J* = 8.5 Hz, 1H), 7.47 and 6.97 (d, *J* = 2.2 Hz, 1H), 6.35–6.21 (m, 1H), 6.13–6.02 (m, 1H), 5.80 and 5.68 (s, 1H), 5.70–5.61 (m, 1H), 5.06 (dd, *J* = 16.9, 1.9 Hz, 1H), 4.92 (dt, *J* = 10.2, 2.2 Hz, 1H), 4.17–3.77 (m, 1H), 3.72 (dd, *J* = 10.1, 3.7 Hz, 1H), 3.23–3.07 (m, 2H), 2.39–2.31 (m, 2H), 2.27–2.12 (m, 3H), 1.81–1.63 (m, 1H), 1.55–1.31 (m, 4H) ppm; ^13^C NMR (CDCl_3_, 101 MHz): δ 173.99, 173.93, 136.91, 135.65, 133.59, 132.93, 132.50, 131.86, 129.62, 129.28, 128.67, 127.77, 126.88, 123.98, 118.74, 114.45, 59.34, 55.99, 38.24, 35.25, 29.37, 28.76, 28.34, 22.80 ppm; ESI-HRMS (positive mode): *m/z* 496.1584/498.1573 (^81^Br) [M + H]^+^, M calcd. for C_26_H_30_BrN_3_O_3_ 496.1594.

**Figure 3:**
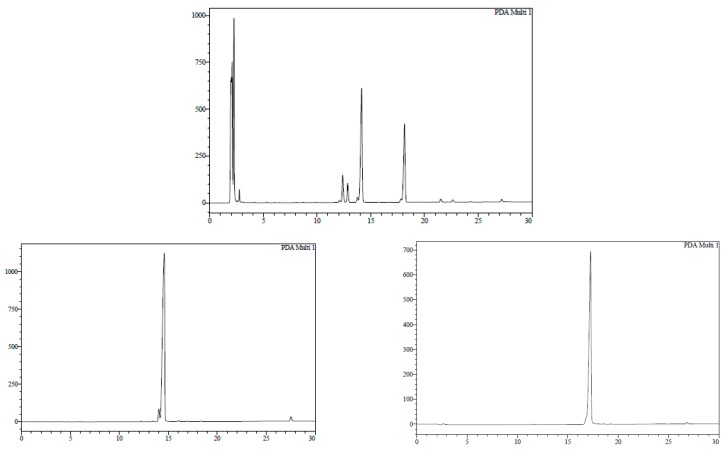
Analytical HPLC traces of crude 16 (top), purified diastereomer 1 (bottom, left) and purified diastereomer 2 (bottom, right). Detection wavelength: 250 nm. Top trace, t_R_ ~ 2–3 min: 15 & *N*,*N’*-diisopropylurea.

RP-HPLC analysis conditions (Figure 3): Jupiter Proteo C_18_ (4 μm, 254 nm, 250 × 4.6 mm) from Phenomenex, 30 → 70% B in 30 min, 1 mL/min, t_R_ = 14.3 min and 18.1 min; purification conditions: Jupiter Proteo C_18_ (10 μm, 254 nm, 250 × 10 mm) from Phenomenex, 40 → 80% B in 30 min, 3 mL/min (t_R_ = 7.5 min and 8.9 min).

### 3.3. Preparation of Oligonucleotides and Conjugates

**Figure 4:**
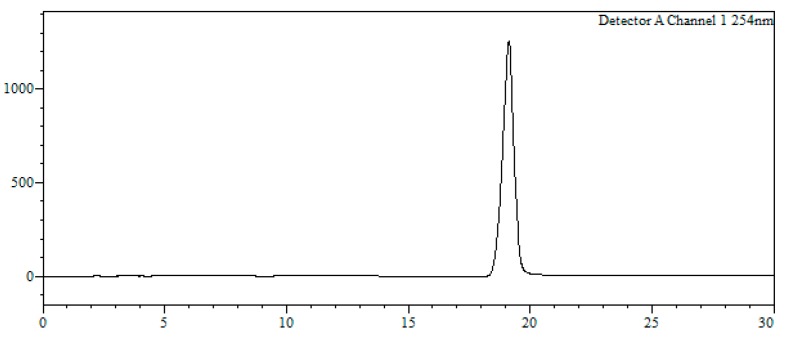
Analytical HPLC trace of oligonucleotide r5’GTTATTCTTTAGAATGGTGC3’. Detection wavelength: 250 nm.

*r5’GTTATTCTTTAGAATGGTGC3’* (2’-*O*Me, PS) (purchased from Avecia, Manchester, UK). HPLC analysis conditions (Figure 4): 0 → 50% B in 30 min, t_R_ = 19.0 min.

MALDI-TOF MS (negative mode, THAP/CA): *m/z* 7060.9 [M − H]^−^, M calcd. for C_218_H_290_BrN_69_O_124_P_19_S_19_ 7053.8.

**Figure 5:**
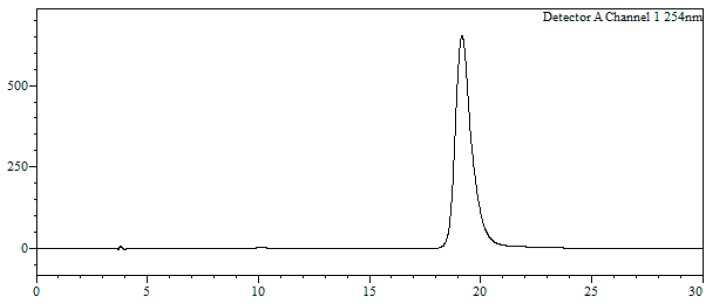
Analytical HPLC trace of oligonucleotide r5’TGTGTACTGATGTAGTTATC3’. Detection wavelength: 250 nm.

*r5’TGTGTACTGATGTAGTTATC3’* (2’-*O*Me, PS). HPLC: Analysis conditions, 0 → 50% B in 30 min, t_R_ = 19.2 min (Figure 5). Purification conditions: 20 → 40% B in 30 min (t_R_ = 9.0 min). Yield (synthesis and purification): 43%.

MALDI-TOF MS (negative mode, THAP/CA): *m/z* 7053.8 [M − H]^−^, M calcd. for C_218_H_290_N_69_O_124_P_19_S_19_ 7053.8.

*Conjugates***19**. Oligonucleotide-resins were automatically assembled at the 1 μmol-scale as stated above (Materials and Methods section). After oligonucleotide elongation was completed and the 5’ end deprotected, phosphoramidite **9** was incorporated (0.1 M solution in anh. acetonitrile, 2 × 10 min coupling) and the resulting phosphite triester sulfurized using the same procedure as in all the other synthesis cycles, which afforded **18**. Final deprotection was carried out under standard conditions (see above). 

**Figure 6:**
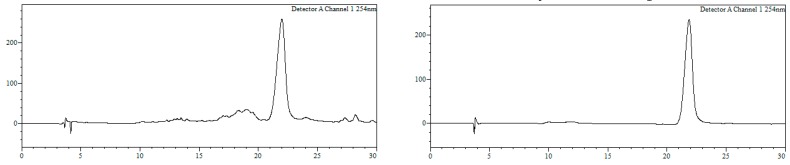
HPLC trace of crude (**left**) and purified (**right**) conjugate **19a**. Detection wavelength: 254 nm.

*Conjugate***19a**. HPLC (Figure 6): Analysis conditions: 0 → 50% B in 30 min, t_R_ = 22.0 min; purification conditions: 0 → 40% B in 30 min (t_R_ = 19.1 min). Yield (synthesis and purification): 36%.

MALDI-TOF MS (negative mode, THAP/CA): *m/z* 7610.5 [M − H]^−^, M calcd. for C_241_H_315_BrN_72_O_128_P_20_S_20_ 7602.8.

**Figure 7:**
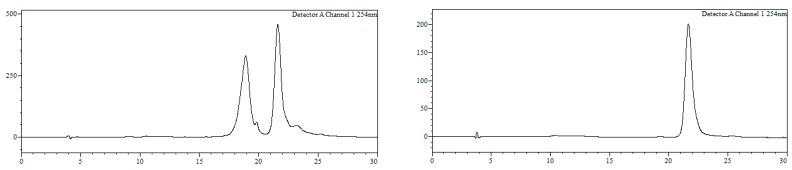
HPLC trace of crude (**left**) and purified (**right**) conjugate **19b**. Detection wavelength: 254 nm. Left trace, t_R_ ~ 19 min: r5’TGTGTACTGATGTAGTTATC3’.

*Conjugate***19b**. HPLC (Figure 7): Analysis conditions, 0 → 50% B in 30 min, t_R_ = 21.7 min; purification conditions: 20 → 40% B in 30 min (t_R_ = 13.8 min). Yield (synthesis and purification): 20%. 

MALDI-TOF MS (negative mode, THAP/CA): *m/z* 7602.1 [M − H]^−^, M calcd. for C_241_H_315_BrN_72_O_128_P_20_S_20_ 7602.8.

*Oligonucleotides***21**. Oligonucleotide-resins were assembled as previously described. The [2,5-dimetylfuran-protected maleimide]-phosphoramidite (see structure in Scheme 5) was subsequently incorporated (0.1 M solution in anh. acetonitrile, 2 × 10 min coupling), which after sulfurization afforded **20**. Treatment with ammonia (as described above) furnished [protected maleimido]-oligonucleotides **21**.

**Figure 8:**
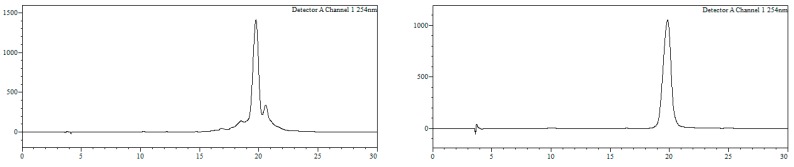
HPLC traces of crude (**left**) and purified (**right**) oligonucleotide **21a**. Detection wavelength: 254 nm.

*Oligonucleotide***21a**. HPLC (Figure 8): Analysis conditions: 0 → 50% B in 30 min, t_R_ = 19.7 min; purification conditions: 20 → 40% B in 30 min (t_R_ = 10.7 min). Yield (synthesis and purification): 26%.

MALDI-TOF MS (negative mode, THAP/CA): *m/z* 7375.1 [M − H]^−^, M calcd. for C_230_H_304_N_70_O_129_P_20_S_20_ 7368.8.

**Figure 9:**
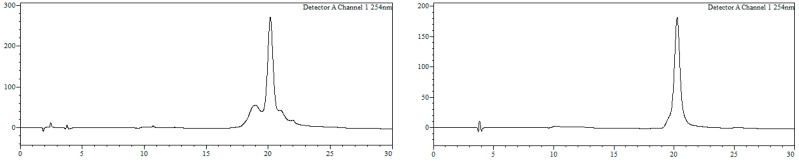
HPLC traces of crude (**left**) and purified (**right**) oligonucleotide **21b**. Detection wavelength: 254 nm. Left trace, t_R_ ~ 19 min: r5’TGTGTACTGATGTAGTTATC3’.

*Oligonucleotide***21b**. HPLC (Figure 9): Analysis conditions, 0 → 50% B in 30 min, t_R_ = 20.2 min; purification conditions: 20→ 40% B in 30 min (t_R_ = 11.3 min). Yield (synthesis and purification): 20%.

MALDI-TOF MS (negative mode, THAP/CA): *m/z* 7369.0 [M − H]^−^, M calcd. for C_230_H_304_N_70_O_129_P_20_S_20_ 7368.8.

**Figure 10:**
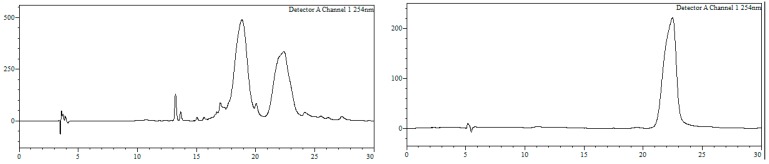
HPLC traces of crude (**left**) and purified (**right**) conjugate **22a**. Detection wavelength: 254 nm.

*Conjugate***22a**. To a solution of **21a** (260 nmol) in MeOH/H_2_O (1:1, 468 µL) a solution of **11** (520 nmol, 52 µL, 10 mM) in MeOH/H_2_O (1:1) was added, and the resulting mixture (final oligonucleotide concentration = 140 µM) heated at 90 °C in a microwave oven for 90 min. Afterwards, MeOH was removed under reduced pressure and the resulting crude purified by HPLC. HPLC (Figure 10): Analysis conditions, 0 → 50% B in 30 min, t_R_ = 22.4 min, purification conditions: 20 → 40% B in 30 min (t_R_ = 14.4 min). Yield (synthesis and purification): 11%.

MALDI-TOF MS (negative mode, THAP/CA): *m/z* 7720.7 [M − H]^−^, M calcd. for C_244_H_318_BrN_73_O_130_P_20_S_21_ 7719.8.

**Figure 11:**
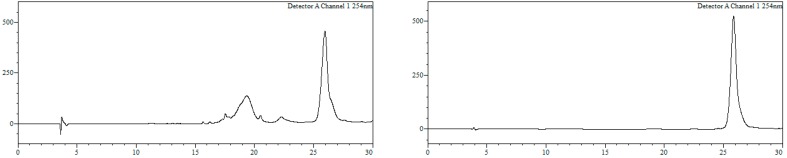
HPLC traces of crude (**left**) and purified (**right**) conjugate **23a**. Detection wavelength: 254 nm.

*Conjugate***23a**. To a solution of **21a** (800 nmol) in MeOH/H_2_O (1:1, 18 mL) **16** was added (4000 nmol, 2000 µL, 2 mM), and the resulting mixture (final oligonucleotide concentration = 0.5 mM) heated at 90 °C in a microwave oven for 90 min. Afterwards, MeOH was removed under reduced pressure and the resulting crude purified by HPLC. HPLC (Figure 11): Analysis conditions, 0 → 50% B in 30 min, t_R_ = 25.9 min; purification conditions: 20 → 40%B in 30 min (t_R_ = 21.3 min). Yield (synthesis and purification): 20%.

MALDI-TOF MS (negative mode, THAP/CA): *m/z* 7772.5 [M − H]^−^, M calcd. for C_250_H_326_BrN_73_O_130_P_20_S_20_ 7767.9

**Figure 12:**
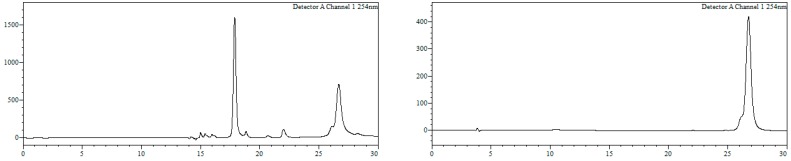
HPLC traces of crude (**left**) and purified (**right**) conjugate **23b**. Detection wavelength: 254 nm. Left trace, t_R_ ~ 18 min: 16.

*Conjugate***23b**. A solution of **21b** (800 nmol) in MeOH/H_2_O (1:1, 19 mL) (final oligonucleotide concentration = 40 µM) was heated at 90 °C in a microwave oven for 90 min. Afterwards, a solution of **16** (4000 nmol) in H_2_O (1 mL) was added and the resulting mixture left stirring for 1 h. Finally, MeOH was removed under reduced pressure and the resulting crude purified by RP-HPLC. HPLC (Figure 12): Analysis conditions, 0→ 50% B in 30 min, t_R_ = 26.7 min; purification conditions: 20 → 40% B in 30 min (t_R_ = 8.5 min). Yield (synthesis and purification): 29%.

MALDI-TOF MS (negative mode, THAP/CA): *m/z* 7758.7 [M − H]^−^, M calcd. for C_250_H_326_BrN_73_O_130_P_20_S_20_ 7767.9.

### 3.4. Luciferase Induction Experiments

Oligonucleotides conjugated to Retro 1 or its derivatives were tested in a splice correction assay using HeLa Luc 705 cells. These cells contain a firefly luciferase expression cassette interrupted by an abnormal intron from thalassemic beta-globin. As a result the cells fail to splice correctly and do not produce luciferase. However, successful intracellular delivery of a splice switching antisense oligonucleotide can correct splicing leading to luciferase expression. Thus this system provides a rapid and convenient assay for the intracellular delivery of oligonucleotides. In the current experiments the HeLa Luc 705 cells were incubated with various concentrations of oligonucleotide and then processed for luciferase induction as described [19]. In some cases there was a brief post-incubation with UNC7938, a small molecule known to enhance oligonucleotide effectiveness [15]. The cells were initially plated at 50,000 per well in 24 well tissue culture plates in DMEM (Dulbecco modified Eagles minimal essential medium) + 10% fetal bovine serum. After overnight incubation at 37 °C and 5% CO_2_, medium was removed and replaced with fresh complete medium containing various concentrations of the oligonucleotides. Cells were then further incubated for 16 h. In some samples this was followed by a 1 h exposure to UNC7938 followed by removal of the compound. After an additional incubation of 2 h, cells were rinsed twice with PBS and then lysed in 1/5 diluted luciferase lysis buffer (Promega, Madison, WI, USA) according to manufacturer’s directions. Luciferase activity was measured using a FLUOstar Omega microplate reader (BMG LABTECH, Cary, NC, USA) and luciferase reagents obtained from Promega.
